# Risk factors for adolescent cannabis use following partial cannabis legalization in Germany: a cross-sectional study in secondary schools

**DOI:** 10.1186/s13011-026-00768-9

**Published:** 2026-10-23

**Authors:** Maike Trümpelmann, Mareike Lüthgen, Annika Burgard, Paul Axt, Henrike Alexandra Faesser, Tobias Jagomast, Markus Weckmann, Daniel Drömann, Folke Brinkmann, Loana Penner, Klaas Frederik Franzen

**Affiliations:** 1 University of Lübeck, University Hospital Schleswig-Holstein, Medical Clinic III (Pulmonology), Campus Lübeck, Lübeck, Germany; 2 Airway Research Center North, Member of the German Center for Lung Research (DZL), 22927 Großhansdorf, Germany; 3 University of Lübeck, University Hospital Schleswig-Holstein, Department of Pediatrics, Campus Lübeck, Lübeck, Germany

**Keywords:** Cannabis, Adolescents, Social media, Risk perception, Prevention, Nicotine, Parental smoking, Legalization

## Abstract

**Background:**

Following Germany’s partial cannabis legalization in April 2024, evidence on adolescent cannabis use risk factors is needed. Social media platforms represent understudied risk environments, and the comparative predictive value of risk perception versus prevention remains unclear. This study aimed to examine determinants of adolescent cannabis use in Germany, including platform-specific social media effects, nicotine advertising exposure, parental smoking, socioeconomic factors, and risk perception.

**Methods:**

A cross-sectional survey of 829 students (age 11 to 19 years) from three secondary schools in northern Germany was conducted between April and July 2024. Cannabis use was measured on a 6-point ordinal scale. Associations were analyzed using ordinal cumulative link models and binary logistic regression, adjusted for age, gender, and school type.

**Results:**

Lifetime cannabis use was reported by 3.8% of younger students (grades 5–7) and 20.3% of older students (grades 8–12), rising from 1.0% at age 13 to 39.1% at age 18. Social media use was strongly associated with cannabis consumption, particularly Instagram (OR = 4.3, 95% CI: 1.79–12.74, *p* = 0.003). Exposure to nicotine advertising on digital platforms increased the odds of cannabis use (OR = 2.02, 95% CI: 1.27–3.20, *p* = 0.003). Additional risk factors included parental smoking in the adolescent’s presence (OR = 2.22–2.78, *p* = 0.001) and higher pocket money (> 50 €/month; OR = 3.0, *p* < 0.001). Perceived health risk of cannabis relative to cigarettes showed the strongest association with cannabis use (Nagelkerke R² = 0.20); adolescents rating cannabis as “much healthier” than cigarettes had substantially higher odds of cannabis use (OR = 22.5, 95% CI: 10.39–49.98, *p* < 0.001). School-based prevention showed no significant association (*p* = 0.70). Cross-substance analyses indicated that lower perceived cannabis risk relative to cigarettes was associated with higher nicotine use.

**Conclusions:**

Social media use and risk perception are key, modifiable factors of adolescent cannabis use. These findings may inform future prevention research focusing on credible risk communication and digital-platform contexts.

## Introduction

Cannabis is the most widely used illicit substance among adolescents worldwide, with lifetime prevalence ranging from 15 to 30% among 15–16-year-olds and exceeding 50% by age 18 in high-prevalence countries [1, 2]. Initiation typically occurs in mid-adolescence [3], underscoring this developmental period as particularly vulnerable.

Following Germany’s partial legalization of cannabis in April 2024, renewed attention to adolescent risk environments is warranted. The Cannabis Act (CanG) permits adults to possess limited amounts of cannabis and cultivate it for personal use, while maintaining a ban on use, possession, and advertising for minors; unlike commercial legalization models in parts of North America, it excludes retail sale and advertising [4, 5]. Evidence from countries that have liberalized cannabis laws indicates that youth prevalence does not uniformly increase after legalization, with strict regulatory frameworks, including advertising restrictions, potency limits, and age verification, potentially mitigating such increases [4, 5]. National surveillance data suggest that adolescent cannabis use in Germany had remained relatively stable in the years preceding legalization [6], providing a reference point for the post-legalization rates reported here.

Digital media environments have become central contexts for adolescent socialization and health-related exposure. Meta-analytic evidence links frequent social media use to higher odds of substance use, particularly on image- and video-centric platforms such as Instagram and TikTok [7, 8]. Content analyses indicate that most substance-related posts portray use positively or normalize consumption [9]. Such environments may influence behavior through normalization processes, reduced perceived harm, peer reinforcement, and indirect marketing exposure [10, 11].

Familial factors also shape adolescent cannabis use. Parental smoking, particularly in the adolescent’s presence, has been identified as a risk factor, potentially via social modeling, reduced perceived harm, and nicotine-related gateway processes [12, 13].

Socioeconomic conditions contribute to cannabis use risk: higher discretionary spending (e.g., pocket money) is linked to elevated substance use, likely reflecting greater access [13, 14]. Cannabis prevalence is also often higher in less academically oriented school tracks [16].

Among the various determinants identified to date, risk perception has emerged as one of the most robust predictors of cannabis use. Systematic reviews consistently demonstrate that adolescents who perceive cannabis as less harmful are more likely to initiate use, consume more frequently, and develop cannabis use disorders [15]. At the same time, emerging evidence points to potential substitution effects: adolescents with strong anti-cannabis attitudes may show higher use of nicotine products, suggesting risk displacement rather than an overall reduction in substance use [17]. In contrast, the effectiveness of school-based prevention programs remains contested. Although meta-analyses report small but statistically significant effects, outcomes appear highly dependent on program quality, duration, interactivity, and implementation fidelity [18–20].

The factors examined in the present study can be situated within a social-ecological framework of adolescent substance use, in which health-related behavior is understood as being associated with influences operating across multiple levels. In this framework, perceived cannabis-related health risk represents an individual cognitive factor; parental smoking reflects the interpersonal and family environment; school type and disposable pocket money capture socioeconomic context; and social media use and nicotine-advertising exposure represent broader media and environmental influences. This framework provides an organizing structure for the predictors examined here while recognizing that, given the cross-sectional design, the direction and causal ordering of these associations cannot be established.

Against this background, the present study addresses key research gaps by examining associated factors of adolescent cannabis use in Germany following partial legalization. Using data from the School*Vape*Science prevention initiative conducted across secondary schools with diverse socioeconomic profiles, we investigated associations between platform-specific social media use, exposure to nicotine advertising, parental smoking, socioeconomic factors, risk perception, and cannabis consumption. By integrating digital, familial, and cognitive factors, this study aims to inform targeted and evidence-based prevention strategies within a rapidly evolving regulatory and media landscape.

## Methods

### Study design and setting

This cross-sectional study was conducted within the framework of the School*Vape*Science project, a combined educational and research initiative focusing on scientific and health literacy and prevention among adolescents. Data collection took place between April and July 2024 in three secondary schools located in Lübeck, Germany, and the surrounding districts. The participating schools were selected to reflect different local contexts, including urban, suburban, and rural settings. Together, they represented three forms of secondary education in the German school system, comprising one academic-track school (Gymnasium) and two comprehensive schools (Gemeinschaftsschulen), one with academic track and one without academic track, thereby capturing socioeconomic heterogeneity. The academic-track school is an academically selective track leading to university-entrance qualification, while the comprehensive school is non-selective and enrolls students of all ability levels. As school-track choice is itself associated with family socioeconomic and educational background, school type served here as a proxy for socioeconomic heterogeneity rather than a direct SES measure. Lübeck is a mid-sized city (approximately 220,000 inhabitants) in northern Germany; schools were selected through existing collaborative relationships with the School*Vape*Science network rather than random or stratified sampling.

The project was implemented using a mixed-format approach. It included (i) a six-week school-based course introducing students to scientific methods, data collection, and health literacy, and (ii) standardized 90-minute preventive teaching sessions delivered to all students at the participating secondary schools. Within this framework, students were involved in the co-development and evaluation of a digital questionnaire assessing substance use, socioeconomic characteristics, and health-related perceptions. Students who participated in the questionnaire development were excluded from the survey sample to prevent overlap between instrument development and data collection.

Participation in the six-week course differed across schools due to organizational structures. At two schools, the course was mandatory, whereas at one school participation was voluntary. No academic credits, grades, certificates, or other incentives were provided for participation in any component of the project.

### Participants

All students attending grades 5 through 12 at the three participating secondary schools were invited to take part in the survey. In the German school system applicable to the study’s region, entry into secondary education typically occurs at ages 10–11 (grade 5). Survey participation was voluntary and anonymous. For analytical purposes, the sample was divided into two age-based groups: a younger cohort including students in grades 5–7 (typically aged 10–13 years) and an older cohort comprising students in grades 8–12 (typically aged 13–19 years). This grouping was chosen to reflect developmental differences and variations in substance exposure risk and is consistent with stratification approaches used in previous European public health surveys on adolescent substance use [21, 22].

### Measures

This study examined one primary outcome (frequency of inhalative cannabis use) as well as a range of predictor variables from four domains: digital media exposure (platform-specific social media use, exposure to nicotine advertising), familial and socioeconomic factors (parental smoking, pocket money, parental education, school type), cognitive factors (perceived health risk of cannabis relative to cigarettes), and prevention exposure (prior participation in school-based cannabis prevention). Age and gender were included as covariates in all models. The operationalization of each variable is described below.

The questionnaire was developed in LimeSurvey and refined through expert review and cognitive pretesting to ensure clarity and age-appropriateness. Two versions were used: a short form for grades 5–7 (28 items) and an extended form for grades 8–12 (40 items including risk perception and advertising exposure).

Frequency of inhalative cannabis use was assessed using the item: “How often do you smoke joints?” Responses were recorded on a 6-point ordinal scale ranging from never (0) to daily use [5]. This measure captured smoked cannabis only; other consumption modalities were not assessed. For binary analyses, responses were dichotomized into no use (never) versus any use (tried at least once to daily).

Social media use was assessed using binary (yes/no) items indicating current use of specific platforms (Instagram, TikTok, YouTube, Snapchat, and others). These items captured any current use of each platform without distinguishing frequency, intensity, or duration of use. These items assessed general platform use only and did not capture exposure to or engagement with cannabis-related content specifically.

Exposure to nicotine product advertising was assessed using binary items indicating any perceived exposure to nicotine-product advertising across multiple channels, including television, print media, and digital platforms (Instagram, TikTok, YouTube, and other social media). As with social media use, these items captured any exposure without differentiating frequency, intensity, or recency of exposure, allowing for analysis of traditional and online advertising exposure.

Parental smoking was assessed using a categorical item distinguishing between no smoking, smoking outside the adolescent’s presence, smoking outdoors in the adolescent’s presence, and smoking indoors in the adolescent’s presence, enabling dose–response analyses.

Age was recorded in years and cross-checked against grade level. Gender was self-reported (male, female, diverse); due to the small number of respondents identifying as diverse (*n* = 10), this category was excluded from multivariable models involving gender as a covariate. As this item captured self-identified gender rather than biological sex, the term “gender” is used consistently throughout this manuscript.

School type was categorized as “Gymnasium” or “Gemeinschaftsschule”. Monthly pocket money was assessed categorically (none, < 10 €, 10–20 €, 20–50 €, 50–100 €, > 100 €). Parental education was recorded separately for each parent using German educational categories. Sports participation and reading behavior were assessed as potential protective factors.

Perceived health risk of cannabis relative to cigarettes was assessed only in the older cohort using a 5-point scale ranging from “much healthier” to “much more harmful.”

Prior participation in school-based cannabis prevention education was assessed using a single dichotomous item (yes/no) asking whether students had previously taken part in any school-based cannabis prevention activity. This item did not differentiate between program types (e.g., skills-based versus information-only approaches), content, duration, or delivery quality.

### Data collection procedures

Data collection was conducted electronically via QR code during designated class periods from April to July 2024. Students completed the questionnaires individually using personal devices or school-provided tablets to reduce peer influence. Research staff were present to resolve technical issues but did not assist with item interpretation to ensure standardized administration. The study design allowed for potential longitudinal follow-up assessments.

In accordance with the approved ethics protocol, written parental/guardian consent was obtained for participants under 18 years, with adolescent assent additionally obtained after age-appropriate information on purpose, voluntariness, and the right to withdraw; participants or older consented directly. The questionnaire was administered anonymously without collection of names or other identifiers, completed individually to reduce social desirability bias, and without teacher presence during completion.

### Statistical analysis

Analyses were performed using R (Version 4.5; R Foundation for Statistical Computing, Vienna, Austria). Continuous variables are summarized as means with standard deviations (SD), and categorical variables as frequencies and percentages.

Ordinal logistic regression using cumulative link models was the primary analytical approach for the 6-level cannabis-use frequency outcome. Binary logistic regression (any lifetime use vs. no use) was additionally performed to facilitate comparison with previous studies and to assess robustness across outcome specifications.

Separate domain-specific multivariable models were fitted for social media use, nicotine-advertising exposure, socioeconomic factors, parental smoking, risk perception, and school-based prevention. Models were adjusted for age, gender, and school type where applicable. No single model including all predictor domains simultaneously was fitted because predictor availability differed across domains and some measures were assessed only in the older cohort.

Complete-case analysis was applied separately within each model, and the corresponding sample size is reported with the respective results. Multicollinearity was assessed using variance inflation factors (VIF), with VIF > 5 considered problematic; no predictor exceeded this threshold. Model fit was assessed using likelihood-ratio chi-square statistics and Nagelkerke pseudo-R².

The proportional-odds assumption was assessed using the Brant test for ordinal models, with χ², degrees of freedom, and p values reported in Section "Results". Odds ratios (ORs) are presented with 95% confidence intervals (CIs). Statistical significance was defined as two-sided *p* < 0.05; results with p values between 0.05 and 0.10 were interpreted cautiously. No correction for multiple testing was applied.

Because only three schools participated, no multilevel or cluster-adjusted analysis was performed; potential underestimation of standard errors due to within-school clustering is acknowledged as a limitation.

## Results

### Study population and baseline characteristics

**Fig. 1:**
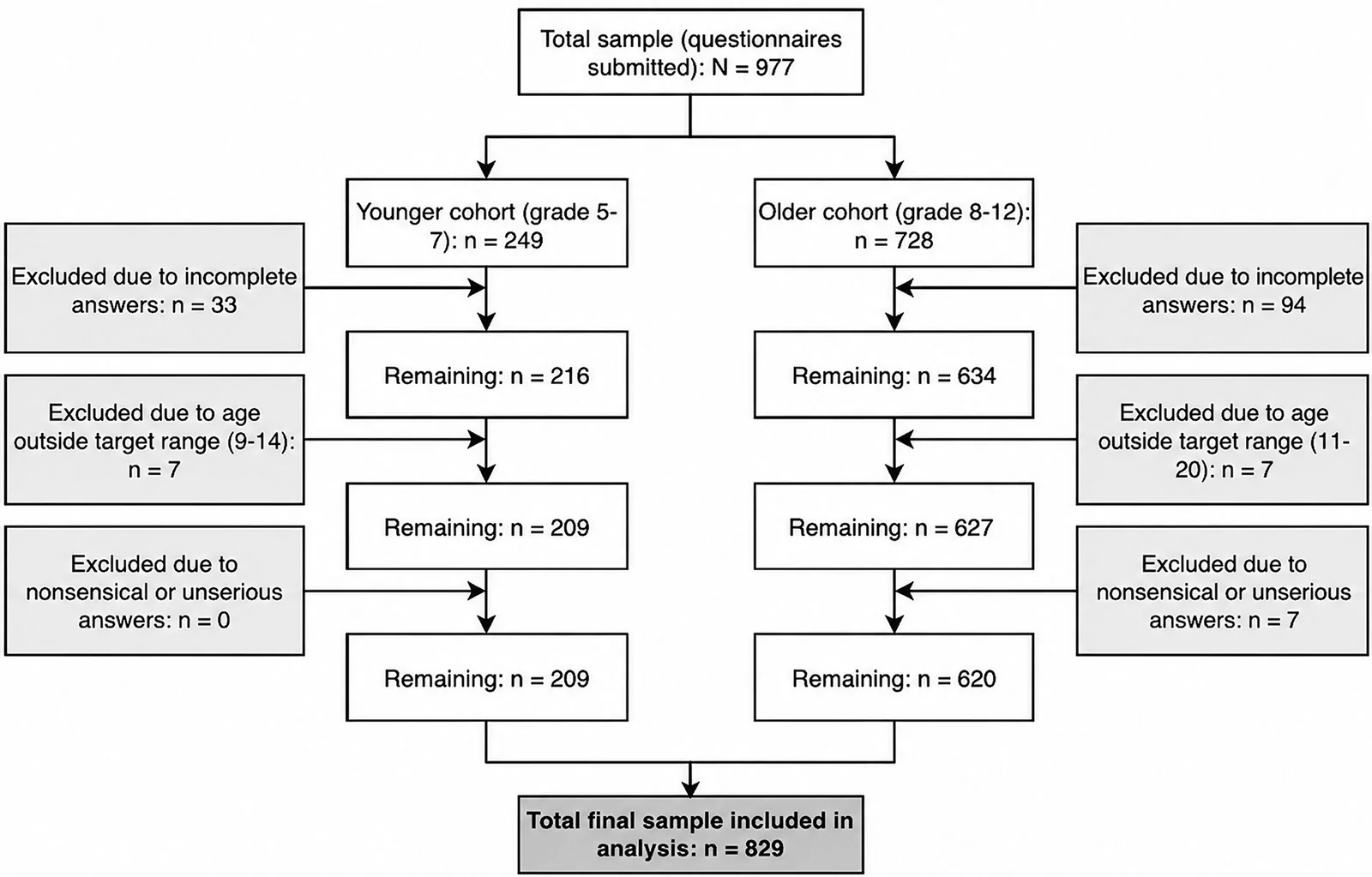
Flowchart

Of the 977 students who initiated the survey, 829 (84.9%) provided complete and valid responses and were included in the final analysis (Fig. 1). The analytical sample comprised 209 students in the younger cohort (grades 5–7; typically aged 10–13 years) and 620 students in the older cohort (grades 8–12; typically aged 13–19 years).

Baseline demographic characteristics by cohort are presented in Table 1. Gender distribution was balanced in both cohorts and was not significantly associated with cannabis use (*p* > 0.05), whereas age showed a strong positive association with consumption frequency (β = 0.53, *p* < 0.001). Social media use was common overall but differed substantially by cohort, with markedly higher usage rates among older students (Table 1).

**Table 1:** Baseline demographic characteristics by age cohort

Characteristic	Younger cohort (grades 5–7)	Older cohort (grades 8–12)
n	209	620
Age range, years	10–13	13–19
Gender, %		
Male	48.8	48.9
Female	49.3	50.2
Diverse	1.9	1.0
School type, %		
Academic track (Gymnasium)	9.1	34.2
Comprehensive (Gemeinschaftsschule)	90.9	65.8
Social media use		
Instagram use, %	42.3	87.2
TikTok use, %	59.3	80.7

### Proportion of inhalative cannabis use

#### Overall proportion of use by cohort

The proportion of cannabis use differed markedly between the age groups. In the younger cohort (grades 5–7; typically aged 10–13 years), 96.2% (*n* = 201) reported never having smoked cannabis, with only 3.8% (*n* = 8) reporting any lifetime use of cannabis.

In contrast, the older cohort (grades 8–12; typically aged 13–19 years) demonstrated a substantially higher proportion of use: 20.3% (*n* = 126) reported any lifetime cannabis use.

Binary logistic regression confirmed a significant positive association between age and cannabis use (OR = 1.71, 95% CI: 1.51–1.94, *p* < 0.001; β = 0.53), indicating that the probability of cannabis use increased with each additional year of age. Table 2 presents the detailed age-specific distribution of cannabis consumption frequency across the total sample population.

**Table 2:** Frequency of inhalative cannabis consumption by age

Age	Inhalative cannabis consumption							
	Never	At least once a lifetime	More than once a month	One to three times a week	More than three times a week	Daily	Total	Reported lifetime use (%)
11	25	1	0	0	0	0	26	3.8
12	57	2	0	0	0	0	59	3.4
13	99	1	0	0	0	0	100	1.0
14	125	9	2	1	0	2	139	10.1
15	166	19	3	1	2	0	191	13.1
16	135	26	6	4	1	1	173	22.0
17	42	14	4	1	1	0	62	32.3
18	42	18	7	0	2	0	69	39.1
19	4	3	1	0	1	1	10	60.0
**Total**	**695**	**93**	**23**	**7**	**7**	**4**	**829**	**16.2**

### Social media platform use and cannabis consumption

Social media use was associated with cannabis consumption, with Instagram showing the strongest and most consistent association among the examined platforms. Descriptively, lifetime cannabis use was substantially more prevalent among Instagram users than non-users (20.6% vs. 2.5%). In ordinal cumulative link models, Instagram use was strongly associated with higher cannabis use categories after controlling for age, gender, and school type (adjusted OR = 4.3, 95% CI: 1.79–12.74, *p* = 0.003; β = 1.5). Dichotomous logistic regression analyses yielded consistent results (adjusted OR = 4.4, 95% CI: 1.84–13.19, *p* = 0.003; β = 1.5).

TikTok use showed weaker and less consistent associations. Cannabis use was reported by 19.1% of TikTok users compared with 6.9% of non-users. TikTok use was significantly associated with higher consumption frequency after adjustment for age, gender, and school type (adjusted OR = 2.0, 95% CI: 1.08–3.96, *p* = 0.034; β = 0.70). In dichotomous models, TikTok use showed a comparatively weaker but still significant association (adjusted OR = 2.1, 95% CI: 1.10–4.09, *p* = 0.030; β = 0.72).

To examine whether Instagram’s association was specific to cannabis or reflected more general substance-related platform effects, effect sizes were compared across substances using ordinal models adjusted for age (to ensure comparability across substances). After age adjustment, Instagram showed a stronger association with cannabis use (OR = 4.03, 95% CI: 1.66–11.57, *p* = 0.005; β = 1.4) than with cigarette smoking (OR = 2.62, 95% CI: 1.57–4.54, *p* < 0.001; β = 0.96) or vaping (OR = 2.04, 95% CI: 1.34–3.15, *p* = 0.001; β = 0.71).

By contrast, TikTok exhibited more uniform associations across substances, with comparable effect sizes for cannabis (OR = 2.14, 95% CI: 1.18–4.15, *p* = 0.016; β = 0.76), cigarettes (OR = 2.56, 95% CI: 1.64–4.11, *p* < 0.001; β = 0.94), and vapes (OR = 3.29, 95% CI: 2.21–5.00, *p* < 0.001; β = 1.19). This pattern indicates that Instagram’s association remains somewhat more pronounced for cannabis-related behaviors after accounting for age, whereas TikTok appears to be similarly associated with multiple substance use categories. The proportional odds assumption was violated in the adjusted Instagram/TikTok model (Brant test: χ² = 79.22, df = 24, *p* < 0.001), driven primarily by gender and Instagram use.

### Nicotine advertising exposure and cannabis use

Ordinal cumulative link models indicated a significant overall association between nicotine advertising exposure across media channels and cannabis consumption frequency (χ² = 31.73, *p* < 0.001; Nagelkerke R² = 0.081; *n* = 620), with a Nagelkerke pseudo-R² of 0.081. The proportional odds assumption was met for this model overall (Brant test: χ² = 51.06, df = 44, *p* = 0.216), with one exception (other social media channels, *p* = 0.003).

#### Traditional media advertising

Exposure to nicotine advertising in traditional media was inversely associated with cannabis use. Television advertising exposure showed an inverse but non-significant association with cannabis consumption frequency (OR = 0.72, 95% CI: 0.46–1.13, *p* = 0.156; β = −0.32). Magazine advertising showed a significant inverse association (OR = 0.49, 95% CI: 0.25–0.94, *p* = 0.039; β = −0.70).

#### Digital media advertising

Exposure via social media platforms other than Instagram, TikTok, and YouTube was associated with higher cannabis consumption (OR = 2.02, 95% CI: 1.27–3.20, *p* = 0.003; β = 0.70). Newspaper advertising showed a significant positive association (OR = 1.96, 95% CI: 1.00–3.77, *p* = 0.045; β = 0.68), differing from the inverse association observed for magazine advertising.

Exposure to nicotine advertising on Instagram was significantly associated with higher cannabis use (OR = 1.80, 95% CI: 1.02–3.23, *p* = 0.045; β = 0.59), whereas TikTok-related advertising exposure was not significantly associated with cannabis consumption (OR = 1.09, *p* = 0.763; β = 0.09). Radio advertising exposure showed a significant inverse association (OR = 0.14, 95% CI: 0.02–0.59, *p* = 0.018; β = −1.94).

### Socioeconomic determinants

#### Pocket money

Monthly pocket money was strongly associated with cannabis consumption (χ² = 103.29, *p* < 0.001; Nagelkerke R² = 0.21; *n* = 603). The model showed a Nagelkerke pseudo-R² of 0.21. The association was non-linear. Using no pocket money as the reference category, lower monthly amounts were associated with lower cannabis consumption, whereas higher amounts were associated with increased use. Receiving 10–20 € per month was associated with lower odds of cannabis use (OR = 0.40, 95% CI: 0.14–1.03, *p* = 0.071; β = −0.91). Similar results were observed for 20–50 € per month (OR = 0.51, 95% CI: 0.27–0.99, *p* = 0.043; β = −0.68). In contrast, higher pocket money amounts were associated with elevated cannabis use, with odds ratios of 3.0 (95% CI: 1.62–5.85, *p* < 0.001; β = 1.11) for 50–100 € per month and 2.8 (95% CI: 1.18–6.47, *p* = 0.019; β = 1.02) for ≥ 100 € per month. Brant test results for this model were not interpretable due to numerous sparse cells.

#### School type

School type was significantly associated with cannabis consumption. Students attending academic-track secondary schools reported lower cannabis use compared with those attending comprehensive secondary schools (OR = 0.47, 95% CI: 0.27–0.78, *p* = 0.004; β = −0.76).

#### Other socioeconomic factors

Several additional socioeconomic indicators were not significantly associated with cannabis use in this sample. These included parental educational level, reading behavior, and sports participation (all *p* > 0.2).

### Parental smoking behavior

Parental smoking was reported by 47.4% of students in the younger cohort and 45.1% in the older cohort; among these, 23.9% and 26.8%, respectively, reported parental smoking in their presence. Parental smoking was significantly associated with adolescent cannabis use, with effect sizes varying by smoking context. Compared with adolescents whose parents did not smoke, parental smoking in the adolescent’s presence showed the strongest associations. Smoking outdoors in the adolescent’s presence was associated with higher cannabis use (OR = 2.78, 95% CI: 1.68–4.57, *p* < 0.001; β = 1.02), and smoking indoors in the adolescent’s presence was also significantly associated (OR = 2.22, 95% CI: 1.05–4.47, *p* = 0.029; β = 0.80). Smoking outdoors without the adolescent’s presence showed a comparable association (OR = 1.88, 95% CI: 1.03–3.33, *p* = 0.035; β = 0.63), while smoking indoors without the adolescent’s presence showed a similarly elevated but statistically non-significant association, likely reflecting the small number of adolescents in this category (OR = 2.52, 95% CI: 0.37–10.25, *p* = 0.249; β = 0.93; *n* = 9). The proportional odds assumption was met for this model overall (Brant test: χ² = 41.22, df = 32, *p* = 0.127), though borderline for the gender covariate (*p* = 0.016).

### Risk perception and school-based prevention

#### School-based cannabis prevention

No association was observed between prior participation in school-based cannabis prevention education and cannabis use. Participation in prevention education showed a small and non-significant association with cannabis consumption (OR = 1.08, 95% CI: 0.71–1.65, *p* = 0.723; β = 0.08).

#### Risk perception and cannabis use

Perceived health risk of cannabis relative to cigarettes showed the strongest association with cannabis use among all examined variables. This analysis was restricted to the older cohort (n = 614). Using the perception of cannabis as “similarly harmful as cigarettes” as the reference category, lower perceived harm was associated with higher cannabis use. Adolescents rating cannabis as ‘much healthier’ than cigarettes showed substantially higher odds of use (OR = 22.50, 95% CI: 10.39–49.98, *p* < 0.001; β = 3.11), and those rating it as ‘somewhat healthier’ also showed elevated odds (OR = 4.50, 95% CI: 2.51–8.22, *p* < 0.001; β = 1.50). No significant associations were observed among adolescents perceiving cannabis as more harmful than cigarettes (both *p* > 0.19). The model including risk perception explained a larger proportion of variance than any other cannabis model in this study (Nagelkerke R² ≈ 0.20). The proportional odds assumption was met for this model (Brant test: χ² = 22.31, df = 20, *p* = 0.324).

#### Cross-substance associations

Cross-substance analyses showed that perceived cannabis risk relative to cigarettes was also associated with nicotine product use. Using the perception of cannabis as “similarly harmful as cigarettes” as the reference category, adolescents rating cannabis as “much healthier” than cigarettes showed higher odds of cigarette smoking (OR = 7.58, 95% CI: 3.92–14.69, *p* < 0.001; β = 2.03) and vaping (OR = 7.43, 95% CI: 3.86–14.41, *p* < 0.001; β = 2.01). Adolescents rating cannabis as “somewhat healthier” showed comparably elevated odds of cigarette smoking (OR = 3.82, *p* < 0.001, β = 1.34) and vaping (β = 1.47, OR = 4.33, *p* < 0.001). No significant differences from the reference category were observed among adolescents perceiving cannabis as more harmful than cigarettes (all *p* > 0.14).

### Summary of key findings

Table 3 summarizes the variables most strongly associated with adolescent cannabis use in this study, ordered by effect size.

**Table 3:** Factors associated with adolescent cannabis use

Predictor	Model type	Effect estimate	95% CI	*p*-value	Nagelkerke pseudo-*R*²
Risk perception (cannabis vs. cigarettes)	Ordinal	β = 3.11 (much healthier); OR = 22.50	10.39–49.98	< 0.001	0.20
Pocket money	Ordinal	β = 1.11 (50–100€); OR = 3.02	1.62–5.85,	< 0.001	0.21
Instagram use	Ordinal	β = 1.5; OR = 4.3	1.79–12.74	0.003	—
Parental smoking (outdoors, in presence)	Ordinal	β = 1.02; OR = 2.78	1.68–4.57	0.001	—
School type (academic track vs. comprehensive secondary school)	Ordinal	β = −0.76; OR = 0.47	CI: 0.27–0.78	0.001	—
Age	Binary	β = 0.53; OR = 1.71	1.51–1.94	< 0.001	—
TikTok use	Ordinal	β = 0.71; OR = 2.01	1.08–3.96	0.034	—
School-based prevention	Ordinal	β = 0.08; OR = 1.08	0.71–1.65	0.723	—

Effect estimates represent adjusted coefficients from the multivariable models. Negative β-values indicate protective associations, whereas positive values indicate risk associations

## Discussion

This cross-sectional study of 829 German secondary school students conducted shortly after partial cannabis legalization in April 2024 identified several factors associated with adolescent cannabis use. Five main findings emerged: (1) proportion of cannabis use increased steeply with age; (2) Instagram use showed the strongest association with cannabis consumption, with larger associations than observed for cigarettes or e-cigarettes (3) perceived health risk of cannabis relative to cigarettes showed the strongest association with use, while school-based prevention education was not associated with consumption; (4) higher pocket money and visible parental smoking were consistently associated with increased use; and (5) lower perceived cannabis risk was associated with higher use of nicotine products. Consistent with the socio-ecological framework outlined in the Introduction, the findings of this study span individual, familial, socioeconomic, and environmental levels, underscoring the multi-level nature of adolescent cannabis use risk.

Cannabis use increased markedly across adolescence, with lifetime use rising from 3.8% in grades 5–7 to 20.3% in grades 8–12, and from 1.0% at age 13 to 39.1% at age 18. This age gradient is consistent with European surveillance data and reflects the concentration of initiation in mid-adolescence [1, 2, 6]. No significant gender differences were observed, contrasting with much of the international literature reporting higher male prevalence [23]. This finding may reflect regional variation or changing gender patterns and warrants further investigation.

Among digital factors, Instagram use showed the strongest association with cannabis consumption, with larger effects than observed for cigarettes or vaping, even after adjustment for age. TikTok associations were weaker and less consistent, reaching significance only in ordinal models. Differences may reflect platform characteristics: Instagram’s visually curated lifestyle content may facilitate normalization and peer modeling [8, 24], whereas TikTok content analyses report lower proportions of positive cannabis portrayals [9].

The magnitude of the Instagram association should nonetheless be interpreted with caution. As platform use was assessed as a simple binary indicator, it may capture broader unmeasured characteristics shared by Instagram users and cannabis users, such as risk-taking propensity, peer group composition, or reduced parental monitoring, rather than a direct platform effect; residual confounding and reverse causality (e.g., adolescents who already use cannabis subsequently engaging more with related content) cannot be excluded. Overall, the findings align with meta-analytic evidence linking social media use to substance use [7], but should be regarded as hypothesis-generating rather than confirmatory of a platform-specific causal pathway.

Exposure to nicotine advertising was associated with cannabis use, with divergent patterns across media channels. Digital nicotine advertising exposure was positively associated with cannabis consumption, whereas exposure via some traditional media channels showed inverse associations. These contrasting findings likely reflect differences in media use patterns rather than causal effects of advertising content. Overall, the results point to the relevance of cross-substance exposure environments, suggesting that nicotine-related marketing may be associated with cannabis use even in the absence of explicit cannabis content. The positive association between digital nicotine advertising exposure and cannabis use is consistent with evidence linking nicotine exposure to subsequent cannabis initiation [25–27]. Exposure to tobacco-related social media content has similarly been associated with increased tobacco use [28], suggesting shared digital exposure environments across substances [29, 30].

Inverse associations observed for television and magazine advertising are unlikely to indicate protective effects and may reflect confounding by media consumption profiles, particularly given the positive association observed for newspaper advertising. Prior research suggests that nicotine-related marketing is associated with cannabis-related behaviors, supporting potential cross-substance priming mechanisms [31, 32].

The non-linear association between pocket money and cannabis use aligns with the economic access hypothesis, whereby greater discretionary resources are linked to increased substance use [14]. Higher monthly amounts may reflect increased spending autonomy and reduced supervision, consistent with prior reviews identifying uncontrolled pocket money as a familial risk factor [13]. It is similarly plausible that adolescents already engaged in substance use seek out additional discretionary income to sustain use, rather than higher pocket money uniformly preceding and enabling initiation.

The association observed for school type (Gymnasium vs. Gemeinschaftsschule; OR = 0.47) is consistent with evidence linking lower academic tracks to higher cannabis use, although this finding should be interpreted cautiously, as only two school types and three schools were included and the association likely reflects selection effects and unmeasured confounding [15].

Parental smoking showed consistent associations with adolescent cannabis use, with effect sizes varying by smoking context. The strongest associations were observed when parents smoked in the adolescent’s presence, followed by smoking outside the adolescent’s presence. These findings suggest that parental smoking may function as an indicator of broader family environments related to substance norms and monitoring, rather than exposure alone [27, 33]. Reciprocal processes may also be relevant at the family level: parental smoking behavior and household norms likely reflect longer-standing family characteristics that predate and remain stable across the adolescent’s development, rather than being directly caused by adolescent cannabis use.

Perceived health risk of cannabis relative to cigarettes showed the strongest association with cannabis use among all variables examined. Adolescents who perceived cannabis as healthier than cigarettes reported substantially higher odds of use, with the strongest association observed among those rating cannabis as “much healthier,” whereas no significant differences emerged among those perceiving cannabis as more harmful than cigarettes. The model including risk perception explained more variance than any other cannabis model in this study (R² = 0.20). In contrast, prior participation in school-based cannabis prevention education was not associated with cannabis use, although this finding should be interpreted cautiously given the binary assessment of prevention exposure, which did not capture program content, duration, or quality. Reverse causality is also plausible: adolescents who already use cannabis may come to perceive it as comparatively less harmful following their own experience, a pattern well documented in longitudinal research on substance-related cognitions [15], rather than low perceived harm exclusively preceding and causing initiation. The cross-sectional design cannot disentangle these directions.

The strong association between risk perception and cannabis use aligns with evidence linking lower perceived harm to increased use and problematic outcomes [13]. Although reverse causation cannot be excluded [15], risk perception remains a central prevention target. The lack of association with prevention education is consistent with evidence indicating modest and heterogeneous program effects depending on implementation quality [18, 19]. Evidence from structured programs and recent interventions suggests that more intensive approaches can reduce initiation, with some evidence of effects persisting into young adulthood [34, 35], effects that are unlikely to be captured by a binary exposure measure [2, 18, 27, 36].

Cross-substance analyses indicated that lower perceived cannabis risk relative to cigarettes was associated with higher use of nicotine products, while no significant differences emerged among adolescents perceiving cannabis as more harmful than cigarettes. This pattern is consistent with broader evidence that cannabis and nicotine use are complementary rather than substitutive at the population level, with strong bidirectional associations reported in meta-analytic evidence [25] and high rates of co-use documented across multiple contexts [24, 35, 37]. Taken together, these results highlight the importance of considering relative-risk perceptions and cross-substance associations in adolescent substance use research and prevention, while underscoring the need for integrated, multi-substance approaches [38].

### Strengths and limitations

Strengths include the relatively large and socioeconomically diverse sample (*n* = 829), timely data collection following partial legalization, use of complementary ordinal and dichotomous models, and integration of digital, familial, socioeconomic, and cognitive correlates. Participatory questionnaire development supported age-appropriateness, and platform-specific social media measures addressed gaps in research relying on aggregated exposure.

Limitations include the cross-sectional design, self-report bias, regional sampling, restriction to inhalative cannabis use, a binary measure of prevention exposure, and potential residual confounding (e.g., mental health, peer use). Age of first cannabis use was not assessed in this survey; as this measure is known to be associated with subsequent consumption patterns and lifetime use, its absence limits our ability to distinguish early-onset from later-onset use trajectories and should be addressed in future studies.

As the outcome measure captured lifetime and current use rather than use timing relative to the legalization date, this study cannot assess whether or how partial legalization affected adolescent cannabis use; the post-legalization timing of data collection should be understood as descriptive context rather than an evaluated exposure.

As only three schools participated, we were unable to formally adjust for potential clustering of observations within schools; unmeasured school-level characteristics may therefore contribute to some of the associations reported, particularly for school type. The purposive, network-based selection of schools further limits generalizability beyond the participating institutions and the broader Lübeck region. Sample sizes also varied across models due to cohort-specific item administration and complete-case handling of missing data, which limits direct comparability of effect estimates across predictor domains.

A small number of participants identifying as diverse gender (*n* = 10) were excluded from multivariable models involving gender as a covariate due to instability in the resulting estimates; findings regarding gender effects therefore pertain to a binary male/female comparison and cannot be generalized to gender-diverse adolescents.

Most predictors were evaluated across multiple model specifications (ordinal and binary; unadjusted and adjusted; full sample and cohort-specific subsamples) without correction for multiple testing, which increases the probability of Type I error. Findings should therefore be interpreted with this in mind, and results close to the significance threshold (e.g., p-values between 0.05 and 0.10) should be regarded as preliminary rather than conclusive.

The proportional odds assumption, evaluated using the Brant test, was violated for the social media model (see Section "Social media platform use and cannabis consumption") and showed a borderline violation for the gender category in the parental smoking model (see Section "Parental smoking behavior"), indicating that the association between certain predictors and cannabis use may not be uniform across all levels of consumption frequency; results from these models should be interpreted with corresponding caution. Additionally, several categorical predictors included sparse cells (e.g., *n* = 9 for parental smoking indoors without the adolescent’s presence), which may have produced unstable effect estimates for these specific categories, as reflected in wide confidence intervals. Several models also showed evidence of quasi-complete separation during diagnostic testing, likely attributable to small cell sizes in certain predictor categories; while this did not affect the primary reported estimates, it further underscores the need for cautious interpretation of categories with limited sample sizes.

## Conclusions

Cannabis use was associated with age, platform-specific social media use, perceived health risk, socioeconomic factors, and parental smoking, with particularly strong associations observed for Instagram use and perceived cannabis risk. Cannabis and nicotine use were associated with shared risk-perception patterns, underscoring the relevance of integrated, multi-substance perspectives in prevention. The findings suggest that future prevention research and program development should consider platform-specific digital contexts, credible risk communication, and the potential role of family and school environments. Longitudinal and experimental studies are needed to clarify temporal relationships and to evaluate targeted prevention approaches in the post-legalization setting.

## Data Availability

The datasets used during the current study are available from the corresponding author on reasonable request.
